# Supplementary material for: Simulated resections and responsive neurostimulator placement can optimize postoperative seizure outcomes when guided by fast ripple networks
Source: Brain Commun. 2024 Oct 14;6(5):fcae367. doi: 10.1093/braincomms/fcae367 (PMC11503960; doi:10.1093/braincomms/fcae367)
Supplement: fcae367_Supplementary_Data [file fcae367_supplementary_data.docx]

**Supplementary Data**

**Simulated resections and responsive neurostimulator placement can optimize post-operative seizure outcomes when guided by fast ripple networks**

Short title: Fast ripple virtual surgery optimize outcome

Shennan Aibel Weiss^1,2,3^, Michael R. Sperling^4^, Jerome Engel Jr^7,9,10,11^., Anli Liu^14,16^, Itzhak Fried^8^, Chengyuan Wu^5,6^, Werner Doyle^15^, Charles Mikell III^17^, Sima Mofakham^17^, Noriko Salamon^12^, Myung Shin Sim^13^, Anatol Bragin^7^, Richard Staba^7^

1. Dept. of Neurology, State University of New York Downstate, Brooklyn, New York 11203, USA
2. Dept. of Physiology and Pharmacology, State University of New York Downstate, Brooklyn, New York 11203, USA
3. Dept. of Neurology, New York City Health + Hospitals/Kings County, Brooklyn, NY, 11203 USA
4. Dept. of Neurology, Thomas Jefferson University, Philadelphia, PA 19107, USA
5. Dept. of Neuroradiology, Thomas Jefferson University, Philadelphia, PA, 19107, USA
6. Dept. of Neurosurgery, Thomas Jefferson University, Philadelphia, PA 19107, USA
7. Dept. of Neurology, David Geffen School of Medicine at UCLA, Los Angeles, California 90095, USA
8. Dept. of Neurosurgery, David Geffen School of Medicine at UCLA, Los Angeles, California 90095, USA
9. Dept. of Neurobiology, David Geffen School of Medicine at UCLA, Los Angeles, California 90095, USA
10. Dept. of Psychiatry and Biobehavioral Sciences, David Geffen School of Medicine at UCLA, Los Angeles, California 90095, USA
11. Brain Research Institute, David Geffen School of Medicine at UCLA, Los Angeles, California 90095, USA
12. Dept. of Neuroradiology, David Geffen School of Medicine at UCLA, Los Angeles, California 90095, USA
13. Dept. of Medicine, David Geffen School of Medicine at UCLA, Los Angeles, California 90095, USA
14. Department of Neurology, NYU Grossman School of Medicine, New York, NY, 10016 USA
15. Department of Neurosurgery, NYU Grossman School of Medicine, New York, NY, 10016 USA
16. Neuroscience Institute, NYU Langone Medical Center, New York, NY, 10016 USA
17. Department of Neurosurgery, State University of New York Stony Brook, Stony Brook, New York 11790, USA

Corresponding author: Shennan Aibel Weiss [shennanweiss@gmail.com](mailto:shennanweiss@gmail.com)

Stony Brook University

Department of Neurology

HSC Rm T12-020

Stony Brook, NY 11733-8121

United States of America

Supplementary Table 1: Patient characteristics in the study cohort. Abbreviations L: left, R: right, N/A: not applicable, ATL: anterior temporal lobectomy, MT: mesial-temporal; MTL: mesial temporal lobe, MTS: mesial temporal sclerosis, SMA: supplementary motor area, TBI: traumatic brain injury, LOC: loss of consciousness, RNS: responsive neurostimulator, VNS: vagal nerve stimulator, SUDEP: sudden unexpected death in epilepsy, @: time to last follow up. Rows colored blue indicate patients with poor spatial sampling (no electrodes recording FR on spikes at a rate > 1 minute), and those colored yellow as patients with incomplete spatial sampling (entire FR generating network resected despite non-seizure free outcome [see methods]). The number of nodes in the FR MI network with a local efficiency > 0 are listed with the patient ID in the first column.

| ID  #FR  MI nodes | | Risk Factor | | MRI | PET  (hypo- metabolic) | iEEG clinical consensus SOZs | | Surgery | | Path. | | Outcome | |  |
| --- | --- | --- | --- | --- | --- | --- | --- | --- | --- | --- | --- | --- | --- | --- |
| 1)  IO01  0 nodes  FR on spike positive | | minor TBI | | Normal | L temporal | L MT | | modified L ATL | | Gliosis | | Engel IA@24 months | |  |
| 2)  IO08  27 Nodes | | hyperten- sive enceph- alopathy | | post L ATL | N/A | L middle temporal gyrus | | modified L temporal lobectomy | | Gliosis | | Engel 1A @48 months | |  |
| 3)  IO18  22 Nodes | | Minor TBI | | Normal | Normal | Right insula, cuneus, inferior and middle frontal gyrus | | R. Frontal lobe | | Gliosis | | Engel IA@24  months | |  |
| 4)  4122 | | None | | Normal | R  temporal | R Inferior temporal gyrus | | modified R temporal lobectomy | | Gliosis | | Engel IA@24 months | |  |
| 5)  4124  4 Nodes | | None | | L MTL  white matter hyperintensity | Normal | R SMA | | R frontal lobe resection | | cortical dysplasia | | Engel IA@24 months | |  |
| 6)  4145  32 Nodes | | None | | Normal | Normal | L cingulate gyrus, medial frontal gyrus, middle frontal gyrus, superior frontal gyrus | | L frontal lobectomy | | cortical dysplasia | | Engel IA@40 months | |  |
| 7)  4166  5 Nodes | | meningitis | | Encephalomalacia | L  temporal | L MT, uncus, superior temporal gyrus, frontal lesion | | L temporal and frontal lobe resection | | gliosis | | Engel IB@42 months | |  |
| 8)  IO12  9 Nodes | | none | | 1 cm pineal cyst | R lateral temporal | L MT | | Modified L temporal lobectomy | | Gliosis | | Engel IIB@24  months | |  |
| 9)  IO05  41 Nodes | | febrile seizures | | prior hippocampal sparing temporal lobectomy | N/A | R anterior cingulate, MT, uncus | | R ATL | | gliosis | | Engel IVB@40  months | |  |
| 10)  453  5 Nodes | | None | | T2 hyper- intensity in R temporal pole > L frontal pole. Inferior part of R temporal pole with blurred gray-white matter border | R temporal | R MT | | R anterior ATL | | Cortical dysplasia IIb | | Engel IA@60 months | |  |
| 11)  456 | | None | | Normal | R  temporal | Bilateral MT, middle temporal gyrus R>L | | modified R ATL | | gliosis | | Engel IVC@48  months | |  |
| 12)  462  11 nodes | | TBI, family history | | left superior temporal gyrus encephalomalacia | L  parieto- occipital | L temporal neocortical, L frontal | | modified LATL  hippocampus sparing | | gliosis | | Engel IV@6 months RNS placed and revised | |  |
| 13)  466  8 nodes | | None | | Normal | L temporal | R fusiform gyrus, superior temporal gyrus, uncus | | R ATL | | MTS | | Engel IB@35 months | |  |
| 14)  473 | | TBI w/ LOC | | L MTS, extra-temporal T2 | L temporal and frontal | L MT, fusiform gyrus, uncus | | L MT Visualase | | N/A | | Engel IIIA@18  months | |  |
| 15)  477  10 nodes | | None | | periventricular nodular heterotopia, right frontal T2 | R  temporal | R MT | | ATL | | gliosis | | Engel IB@31  months | |  |
| 16)  479 | | TBI w/ LOC | | Encephalomalacia | R temporal | R insula, bi- lateral middle temporal gyrus, superior temporal gyrus | | modified R ATL | | gliosis | | Engel IVB@33  months | |  |
| 17)  469  8 nodes | | TBI | | left MTS | normal | L MT | | L ATL | | gliosis hippocampal sclerosis | | Engel IIIA@63  months | |  |
| 18)  IO21 | None | | Prior R. ATL | | N/A | | Right orbitofrontal cortex. | | R. Frontal lobe | | hippocampal sclerosis, cortical dysplasia | | Engel IVB@24  months | |
| 19)  4110  10 nodes | encephalitis | | Encephalo- malacia | | Normal | | L inferior frontal gyrus, insula, MT | | L temporal lobe and insula resection | | Gliosis | | SUDEP  @6 weeks | |
| 20)  IO23  20 nodes | Significant head injury with LOC | | Left temporal T2 hyperintensity with mild enhancement | | N/A | | Bilateral MT, right lateral temporal | | L temporal lobectomy, anterior thalamic DBS | | Gliosis | | Engel IVB@24  months | |
| 21)  IO13  10  nodes | None | | R parietal lobe resection | | R parietal and R occipital | | R insula, pre- cuneus, middle occipital gyrus, superior parietal lobule, superior occipital gyrus, superior temporal gyrus, middle temporal gyrus | | R parietal | | gliosis | | Engel IIIA@18  months | |
| 22)  IO15  14 nodes | None | | L posterior fossa arachnoid cyst, R ATL | | R  temporal | | L MT, R cingulate, post. cingulate, mesial frontal, precuneus | | R anterior cingulate thermal ablation | | gliosis | | Engel IVB@36  months | |
| 23)  IO19  23 nodes | None | | Prior R parietal resection | | R parietal and occipital hypometabolism | | R parietal lobe | | R. Parietal lobe resection | | Gliosis | | Engel IVB@36  months | |
